# Accumulation and Toxicity of Superparamagnetic Iron Oxide Nanoparticles in Cells and Experimental Animals

**DOI:** 10.3390/ijms17081193

**Published:** 2016-08-19

**Authors:** Greta Jarockyte, Egle Daugelaite, Marius Stasys, Urte Statkute, Vilius Poderys, Ting-Chen Tseng, Shan-Hui Hsu, Vitalijus Karabanovas, Ricardas Rotomskis

**Affiliations:** 1 Biomedical Physics Laboratory of National Cancer Institute, Baublio 3B, LT08406 Vilnius, Lithuania; greta.jarockyte@nvi.lt (G.J.); e.daugelaite@gmail.com (E.D.); marius.stalnionis@nvi.lt (M.S.); urtestatkute@gmail.com (U.S.); vilius.poderys@nvi.lt (V.P.); vitaliljus.karabanovas@vgtu.lt (V.K.); 2 Institute of Polymer Science and Engineering, National Taiwan University, No. 1, Roosevelt Road Sec. 4, Taipei 10617, Taiwan; bbam1986@hotmail.com (T.-C.T.); shhsu@ntu.edu.tw (S.-H.H.); 3 Department of Chemistry and Bioengineering, Vilnius Gediminas Technical University, LT-10223 Vilnius, Lithuania; 4 Biophotonics group of Laser Research Centre, Vilnius University, Sauletekio 9, c.3, LT-10222 Vilnius, Lithuania

**Keywords:** magnetic nanoparticles, SPIONs, iron oxide, cellular uptake, MRI-optical dual imaging, optical biopsy of tissues cells, multifunctional cancer diagnostics

## Abstract

The uptake and distribution of negatively charged superparamagnetic iron oxide (Fe_3_O_4_) nanoparticles (SPIONs) in mouse embryonic fibroblasts NIH3T3, and magnetic resonance imaging (MRI) signal influenced by SPIONs injected into experimental animals, were visualized and investigated. Cellular uptake and distribution of the SPIONs in NIH3T3 after staining with Prussian Blue were investigated by a bright-field microscope equipped with digital color camera. SPIONs were localized in vesicles, mostly placed near the nucleus. Toxicity of SPION nanoparticles tested with cell viability assay (XTT) was estimated. The viability of NIH3T3 cells remains approximately 95% within 3–24 h of incubation, and only a slight decrease of viability was observed after 48 h of incubation. MRI studies on Wistar rats using a clinical 1.5 T MRI scanner were showing that SPIONs give a negative contrast in the MRI. The dynamic MRI measurements of the SPION clearance from the injection site shows that SPIONs slowly disappear from injection sites and only a low concentration of nanoparticles was completely eliminated within three weeks. No functionalized SPIONs accumulate in cells by endocytic mechanism, none accumulate in the nucleus, and none are toxic at a desirable concentration. Therefore, they could be used as a dual imaging agent: as contrast agents for MRI and for traditional optical biopsy by using Prussian Blue staining.

## 1. Introduction

Since the first contrast medium for magnetic resonance imaging (MRI) was developed [1,2,3], researchers have kept looking for advanced materials and synthesis methods that could be applied in MRI. MRI is an important tool in medicine, offering detailed spatial resolution and soft tissue contrast without the use of ionizing radiation or potentially harmful radiotracers [4,5]. MRI is a well-established but still growing in availability non-ionizing method of tomographic imaging for diagnostics of various diseases including oncological pathologies [6]. At the moment, there are two main compounds used. Iron oxide based agents that provide “negative” contrast in images and gadolinium based agents that account for the “positive” contrast. “Negative” contrast agents are known for creating strong local magnetic field inhomogeneity that influence bypassing water molecules and induce their rapid T2 and T2* relaxations, which appear as a signal loss in MR images of lesions corresponding to iron oxide accumulation [7,8].

The development of nanoparticles for use in biomedicine has shown great progress over the past two decades, and has been tailored for use as contrast enhancement agents for imaging. Magnetic nanoparticles (MNPs), with their unique magnetic properties and controllable sizes, are being actively investigated as the next generation of magnetic resonance imaging contrast agents.

MNPs possess useful properties for a variety of life sciences-related applications, comprising both basic and clinical research [9,10]. A class of nanocompounds that can be manipulated using a magnetic field has been tailored for use as enhancement agents for imaging, drug delivery vehicles, and, most recently, as a therapeutic component in initiating tumor cell death in magnetic and photonic ablation therapies [11]. Iron oxide MNPs with nanocrystaline magnetite (Fe_3_O_4_) cores have great potential for use in oncology due to their biocompatibility, biodegradability, facile synthesis, and ease with which they may be tuned and functionalized for specific application [10]. Spherical iron oxide MNPs will exhibit supermagnetic behavior (a property that is exploited to enhance contrast in MRI). Typically, supermagnetic iron oxide nanoparticle (SPION) conjugates are comprised of a magnetite core providing inherent contrast for MRI and a biocompatible coating that provides ample functional groups for conjugation of additional tumor targeting and therapeutic moieties. SPIONs provide negative (hypointense) contrast by darkening T2 and T2*-weighted images in regions of interest (ROIs) corresponding to uptake areas of SPIONs.

Ferrous or ferric oxide is the main constituent of magnetic particles, although metals such as cobalt and nickel are used in other fields of application. While SPIONs have historically been used primarily for negative contrast enhancement by darkening T2*-weighted images, they may also be customized to provide positive contrast enhancement in T1-weighted scans [12,13]. Nanoparticles with gadolinium (Gd) complexes are known in MR imaging T1 contrast material, although their sensitivity is relatively low [2]. In addition, the side effects related to Gd, especially in patients with kidney problems, demand the development of more superior, safer substances [14,15]. There are overall desirable features of a “perfect” contrast agent that are still not achieved yet and comprised of: easy administration, nontoxicity, stability of a compound, selectivity, sensitivity, quick elimination from the body after the imaging is complete, minimal to no side effects, and cost-effectiveness. SPIONs offer an advantage over Gd-based agents due to both being nontoxic and superior in providing T1 contrast. For instance, a SPION formulation has been developed exhibiting a twofold improvement of T1 contrast enhancement as compared to a commercial Gd-based clinical standard [13]. Different studies have reported from very small to no toxicity of iron oxide nanoparticles [16,17]. SPIONs have some specific properties such as superparamagnetism, high field irreversibility, high saturation field, extra anisotropy contributions or shifted loops after field cooling [18]. Due to these properties, the particles no longer show magnetic interaction after the external magnetic field is removed. Progress in the field of nanotechnology has led to selective tumor markers, connecting to a variety of complex organic polymers and biomolecules [19,20]. MR contrast agents that are composed of the SPION core can be modified with organic substances (Dextran, PEG) to improve their stability in aqueous solutions and add efficiency to the delivery of the nanoparticles to the tumor site [21]. MNPs can also be used for cancer therapy: hyperthermia therapy and anticancer drug delivery. These two methods could be combined: hyperthermia-based drug delivery is therapy in which an anticancer drug is delivered to the tumor by an external magnetic field, and the drug molecule is then released due to heating [22].

Some formulations of magnetite-based NPs have already gained approval for use in humans as iron deficiency therapeutics and as MRI contrast agents by the Food and Drug Administration (FDA) (e.g., FerahemeR (AMAG Pharmaceuticals, Waltham, MA, USA), Feridex I.V.R (AMAG Pharmaceuticals), and GastromarkR (AMAG Pharmaceuticals)) [11]. Up to this date, iron oxide nanoparticles have been approved for clinical use as liver imaging agents [9]. Various SPIONs were investigated as new MRI contrast agent last decay. For about two decades, MNPs have been used as FDA-approved contrast agents (Dextran-coated EndoremR (Guerbet, Paris, France), Feridex I.V.R) [21] in MRI for the detection of liver pathologies [23]. Recently, a novel formulation of SPIONs coated with a carboxylated shell (FerumoxytolR (AMAG Pharmaceuticals)) has been FDA approved for the treatment of iron deficiency anemia in adults with chronic kidney disease [23]. A wide variety of MNPs are being tested for the in vitro labeling of cultured stem cells and their subsequent in vivo tracking by MRI after transplantation. Several of them are undergoing clinical trials [24,25].

However, the translation of these NP formulations to use in medical clinic fails at a very high rate as can be corroborated with the relative dearth of NPs employed for use in humans [26,27]. Specifically for iron oxide NPs, only a handful of formulations have been approved by FDA, and even the most recent magnetite-based NP to be approved, FerahemeR, does not have intended use as an MRI agent or cancer therapeutic [28]. Other iron oxide NP formulations, although approved by the FDA, have stopped being marketed by their manufacturers (e.g., FeridexI.V.R, and GastromarkR) [11]. Currently, no functionalized SPIONs are used in humans.

Nevertheless, despite the obvious impact on MR images, the adverse effect of contrast and artifacts from the magnetization are also factors in determining SPION contrast deficiencies. The darkening of MR images resulting due to SPIONs accumulation in tissue may confuse the clinical diagnosis, judging by the T2 MRI signal. This signal can often be confused with the signal that distinguishes bleeding from the place of metal calcium, derivatives or residues [29].

This indicates that there is still a need for investigation of SPIONs to gather more knowledge about mechanism of their accumulation and biodistribution in cell cultures and in vivo, and revitalization of characteristic appearance on preclinical MRI images [30]. There are not any precise investigations in which the same SPIONs would be used for experiments with cells and continuing on animals. Lack of information of loading capacity and of control over the biodistribution of SPIONs, insufficient evidence about distribution and clearance from the injection site, and migration in the tissue of experimental animals are the major issues holding back their clinical translation.

In this study, we aimed to investigate the accumulation of SPION nanoparticles in embryonic fibroblasts of mice (NIH3T3), their effect on proliferation and viability of cells, to examine the MRI signal intensity versus the SPION concentration, and to evaluate MR signal of SPION injection sites in Wistar rats. For iron visualization in cells and tissues with optical microscopy, Prussian Blue Cell Staining was used. MNP accumulation in experimental animals was visualized by the MRI method.

Even though SPIONs were specifically developed for use as MRI contrast agents, recent efforts have been made to incorporate additional possibilities to enable complementary imaging modalities. MRI has exceptional spatial resolution but lacks sensitivity. Optical imaging is relatively inexpensive and very sensitive but cannot penetrate deep into all tissues due to the attenuation and to scattering of light. Thus, in the study, we are presenting the evidence of combinations of these imaging modalities providing the anatomical resolution (MRI) and molecular sensitivity (Prussian Blue Cell Staining) needed for accurate diagnoses.

## 2. Results

### 2.1. Characterization of Nanoparticles

**Figure 1:**
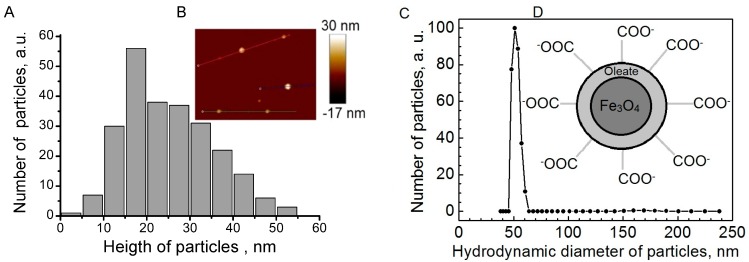
Topographic atomic force microscopy (AFM) image of Fe_3_O_4_ magnetic nanoparticles dispersed on mica surface (**B**), particle height histogram (**A**), hydrodynamic size distribution (**C**) and schematic picture (**D**) of Fe_3_O_4_ magnetic nanoparticles.

Fe_3_O_4_ nanoparticles (NPs) (Figure 1D) were prepared by chemical co-precipitation. After formation of Fe_3_O_4_ NPs, NPs were washed by centrifugation and re-dispersion in distilled water for three times. Sodium oleate (1.5 g dissolved in 50 mL distilled water) was added to the Fe_3_O_4_ NPs under vigorous stirring for 30 min, and the excess surfactant was removed by dialysis so there was no free surfactant.

Topographic atomic force microscopy (AFM) images of Fe_3_O_4_ nanoparticles are shown in Figure 1B. On the surface of mica, single particles similar in size can be seen. There are no aggregates of particles on the surface. AFM measurements showed that height of Fe_3_O_4_ nanoparticles varies from 10 to 50 nm. Particle height distribution obtained from AFM measurements (Figure 1A) reaches a maximum at 15–20 nm. Hydrodynamic size measurements revealed that the diameter of particles in solution is approximately 50 nm (Figure 1C). As it should be expected, measured hydrodynamic radius is slightly larger than the diameter of nanoparticles measured with AFM. Hydrodynamic diameter shows the diameter of the inorganic core with coating material and the layer of solvent attached to the particle as it moves under the influence of Brownian motion. While estimating size by AFM, the hydration layer is not present; hence, only information only about the inorganic core is obtained. However, results obtained by both techniques are in a good agreement. Zeta potential of magnetic nanoparticles in prepared solutions is about −34.34 ± 1.12 mV. This indicates that solution of nanoparticles should be colloidally stable. Solution is considered colloidally stable when zeta potential is less than −30 mV or more than 30 mV [31]. Fe_3_O_4_ nanoparticles from different batches had a similar size range. The average zeta potential was in the range from −50 to −35 mV.

### 2.2. Accumulation and Toxicity of Nanoparticles in Live Cells

**Figure 2:**
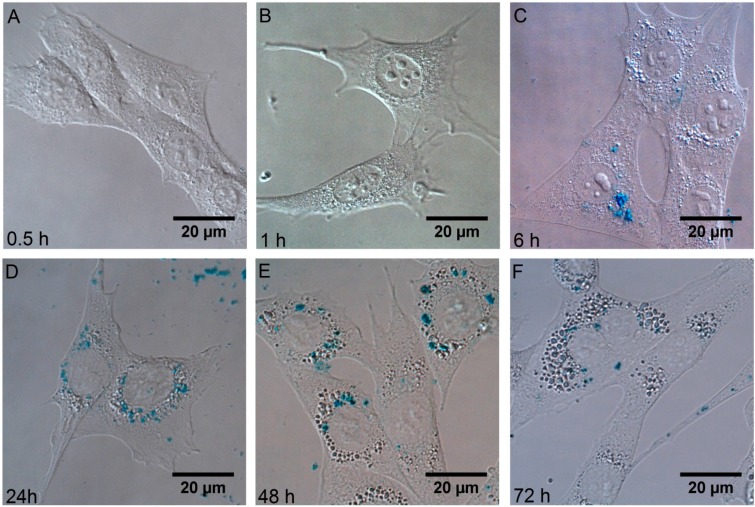
Fixed NIH3T3 cells after 0.5–72 h of incubation with 65 ng/mL of Fe_3_O_4_ (stained with Prussian Blue) (**A**–**F**). The accumulation was observed using bright-field microscope equipped with digital color camera.

Accumulation and biological effects of Fe_3_O_4_ nanoparticles were investigated. An accumulation of particles depends on incubating time. After incubating cells with magnetic nanoparticles for less than 6 h, almost no intracellular accumulation was observed (Figure 2A,B). There were only several cells, which were able to internalize nanoparticles during six hours of incubation, and particles are accumulated through the whole cytosol (Figure 2C). There also were not nanoparticles adherent to cytoplasmic membrane. These results show that Fe_3_O_4_ nanoparticles do not have a strong affinity to plasma membrane. During short incubation times, nanoparticles could be attached to the membrane of the cells, but these nanoparticles were easily washed out before imaging. In comparison with quantum dots, which accumulate in cell membrane structures after 30–60 min of incubations [32], there are not any magnetic nanoparticles adhered to the plasma membrane after the same time of incubation (Figure 2A,B). After longer times of incubation, particles were observed in all cells, localized in the perinuclear region (Figure 2D–F). Due to SPION coatings and relatively large size of whole nanoparticles, the membrane of the cell needs more time to initiate the process of endocytosis. Magnetic nanoparticles have not accumulated in the nucleus of cells. The same results are obtained after NIH3T3 was incubated with quantum dots for 24 h [32].

After 48 or more hours of incubations, a few new structures (large vesicles, blebs or lipid droplets) were observed in the perinuclear region (Figure 2E,F) According to the literature, magnetic nanoparticles might induce formation of lipid droplets [33]. Morphological changes of cells require investigation of the viability of cells after incubations with Fe_3_O_4_ nanoparticles.

**Figure 3:**
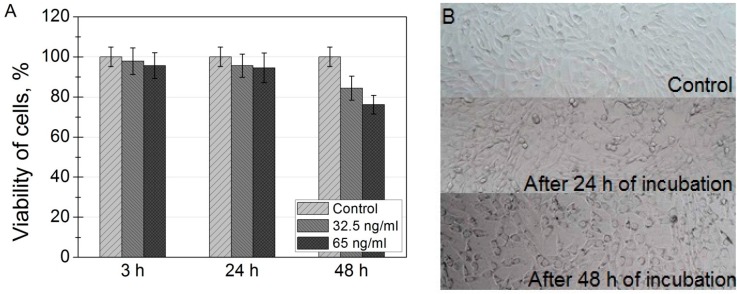
(**A**) viability of mouse embryonic fibroblasts NIH3T3, incubated with SPIONs for 3, 24 and 48 h. Toxicity of nanoparticles was investigated using XTT cell viability assay; (**B**) XTT plate images of NIH3T3 cells incubated with 32.5 ng/mL of SPION nanoparticles for 0, 24 and 48 h.

For assessing cell viability, cells were incubated with two different concentrations of magnetic nanoparticles. No cytotoxicity was observed when cells were incubated with SPIONs for 3 h and 24 h: cell viability remains approximately 95% (Figure 3A). However, a slight decrease of viability was observed after 48 h of incubation. XTT is an indirect method of viability investigation, meaning that, after 48 h of incubation, it decreases the rate of mitochondrial activity. In Figure 3B is shown XTT plate images of NIH3T3 cells after incubation with nanoparticles.

### 2.3. MR Signal Intensity versus the Fe_3_O_4_ Concentration

**Figure 4:**
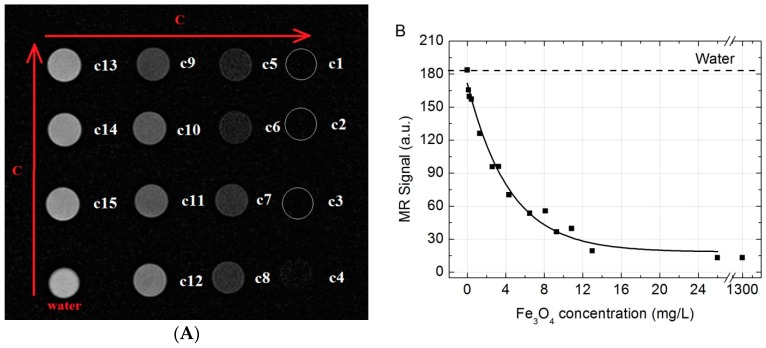
(**A**) T2W MR coronal slice images in vitro of different concentrations of Fe_3_O_4_ dissolved in water (from c1 (1300 mg/L) to c15 (13 mg/L)); (**B**) T2W MR signal intensity plot of aqueous suspensions of Fe_3_O_4_ versus the concentration in the solution. Dotted line marks water MR signal.

T2-weighted magnetic resonance (T2W MR) coronal slice images of different concentrations of Fe_3_O_4_ nanoparticles water solutions are shown in Figure 4A. The MR signal intensity versus the different concentrations in solutions of Fe_3_O_4_ are shown in Figure 4B. As shown in Figure 4, a negative enhancement for MR signal was observed for all the tubes when compared to water (dotted line). Starting from the lowest concentration, the reduction of MRI signal was increasing corresponding to the increase of the SPION concentration up to 25 mg/L. At the concentration of 25 mg/L (c3) or higher no MRI signal could be observed at all (absolute suppression). Therefore, it can be concluded that the concentration below 25 mg/L would be enough to provide the “negative” T2 contrast in MR imaging.

### 2.4. The Migration of SPIONs from the Injection Site

**Figure 5:**
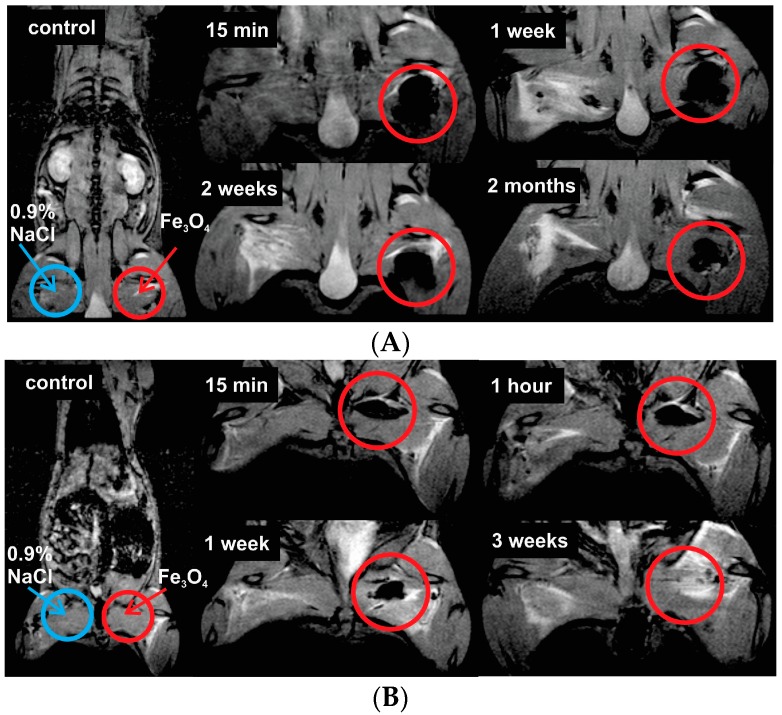
T2W MR coronal slice images of injection site at different time moments after intramuscular injection at dose of (**A**) 520 µg Fe_3_O_4_/kg (in the upper figure) and (**B**) 20.8 µg Fe_3_O_4_/kg (in the lower figure) in the rat left hind paw (red circles). The arrows marks injection site of physiological saline and Fe_3_O_4_ nanoparticles solution.

T2W MR coronal images of the injection site at the different time moments after intramuscular injection of 520 µg Fe_3_O_4_/kg (high dose) in the rat left hind paw (red circles) shown in Figure 5.

**Figure 6:**
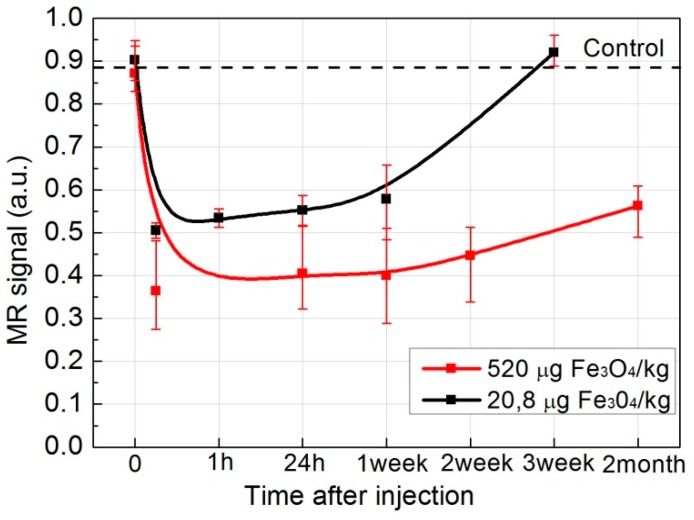
Relative MR signal intensity at the injection site versus time at the different doses of Fe_3_O_4_ (red curve—520 µg Fe_3_O_4_/kg, black—20.8 µg Fe_3_O_4_/kg).

As shown in Figure 5A, a negative enhancement for MR signal at the injection site was observed in all of the images (black areas in red circles) compared with the control image. The decrease over time was observed, implicating that Fe_3_O_4_ nanoparticles were slowly cleared from the injection site. Although, at high doses, even at two months, the Fe_3_O_4_ nanoparticles have not been completely eliminated from the injection site. However, after the injection of the low dose of nanoparticles (20.8 µg Fe_3_O_4_/kg), SPIONs were fully cleared from the injection site within three weeks (Figure 5B). Figure 6 shows relative MR signal intensity versus time at the injection site (Figure 5, red circles) with different doses of Fe_3_O_4_. In the case of low dose injection, the signal intensity by the third week after the injection was equal to control signal intensity (Figure 6, black curve), thus indicating that the low concentration SPIONs were fully metabolized from the injection site. It has been reported and cannot be ignored that exposure to SPION has been associated with significant toxic effects such as inflammation or infection [34,35].

## 3. Discussion

Lately, SPIONs have been attracting attention as new and perspective MRI contrast agents and anticancer drug carriers. However, there are not any precise investigations of accumulation of SPIONs in cells. Most of the evidence of SPION accumulation in cells is electron microscopy and phase contrast images in which only black dots could be observed. However, it is incorrect to state that these black dots are MNPs because they could be lipid droplets, which are induced after incubating cells with magnetic nanoparticles [33]. Moreover, phase contrast images do not give information about MNPs localization in cells because images of MNPs strongly depend on the focal plane. When the focal plane is changed, black dots, which we assumed were MNPs, became white bubbles, which look like vesicles. In our investigation, we used iron staining, which is used for histological samples to detect the presence of iron in biopsy specimens, such as in bone marrow samples [36].

Confocal fluorescence images taken after incubation of the cells with quantum dots show evolution of the distribution pattern and transport vesicles of carboxyl-coated quantum dots in the cells: phase 1—adherence to the cell membrane; phase 2–formation of granulated clusters spreading in the cytoplasm; phase 3—localization of granulated clusters in the perinuclear region; and phase 4—formation of multivesicular body-like structures and their redistribution in the cytoplasm [32]. In comparison with quantum dots, which accumulate in cell membrane structures after phase 1, there are not any magnetic nanoparticles adhered to plasma membrane at any time during incubation (Figure 2A,B). These results show that SPIONs do not have affinity to plasma membrane of the cells. The granulated pattern of the Prussian Blue absorption in NIH3T3 cells after incubation, indicates that the SPIONs were trapped in vesicular structures. They localized in the perinuclear region, and no SPIONs were detected in the nucleus of the cell. Zhu et al. used Prussian Blue staining to detect SPIONs in mammalian cells; however, they did not analyze the localization of SPIONs in cells [37]. The viabilities of NIH3T3 cells indicate good biocompatibility of SPIONs for potential in vivo imaging. These results coincide with previously reported others investigations of toxicity of SPIONs in different cell lines [38].

SPIONs show significant signal reduction with increasing of SPION concentration in the T2-weighted MRI. Similar results were also reported in [39,40]. Although the in vitro dispersion behavior and imaging performance of nanoparticles are different from those in vivo MRI, the brightening of the image spots of the nanoparticles from the injection site through time indicates an excretion, even though, when it comes to relatively large concentrations of the SPIONs, a rather slow excretion from the injection site is observed. The migration of SPIONs from the injection site was investigated by analyzing T2*W MR coronal slice images at different time moments after intramuscular injection (Figure 5 and Figure 6). When concentration of injected SPIONs solution was high (650 mg/L), only a slow removal of nanoparticles from the injection site was observed. However, the lower concentration (26 mg/L) of nanoparticles was completely eliminated from the injection site within three weeks. The former can be attributed to the aggregations of nanoparticles in vivo and could cause false positive findings because of the artifacts; this study suggests the potential of such Fe_3_O_4_ nanoparticles as an effective T2-weighted MRI contrast agents for tumor diagnosis.

At the moment, there are still obstacles to overcome until the SPIONs find their niche in the algorithm of clinical routine. We still need to investigate the better biodistribution and excretion pathways of the nanoparticles, as well as to determine the optimal ways of synthesis to achieve a specific purpose. In addition, the experiments in cells and in animals to this date have not yet been shown to translate well from one technique to another. However, this is very important, when thinking about the clinical application of the SPIONs. Adding the staining method of iron used in this study turns SPIONs into dual imaging tracers, for visualization by the means of MRI and optical imaging without the requirement for invasive methods. In contrast to MRI, optical imaging methods such as Prussian Blue staining have relatively good sensitivity but suffer from low tissue penetration depths. Various nanomaterials such as quantum dots (QDs) and SPIONs have been developed for biomedical applications. QDs for example have good photoemission and photostability characteristics. However, QDs, just like Gaddolinium, have been shown to have cytotoxic effects. Each imaging modality has its own advantages and disadvantages. By combining different modalities of imaging methods can compensate for the disadvantages of a single imaging modality.

Another already mentioned problem is that there are not any papers about investigations of the same SPIONs both in cells and in animals. Our work was a continuous study of SPIONs’ physical properties, accumulation in cells, and biological effect for cells, in addition to measurements of MRI signals of SPIONs in vitro and in vivo. Furthermore, better theoretical in vitro and animal models will help to predict the optimal MNP formulations for humans. As our understanding of MNP behavior in the biological environment improves, we are optimistic that integrated MR and optical imaging with SPIONs will push forward the clinical use of SPIONs.

## 4. Materials and Methods

### 4.1. Synthesis of Fe_3_O_4_ Nanoparticles

Fe_3_O_4_ nanoparticles were synthesized according to previously reported procedure [36]. In addition, 8.95 g FeCl_2_·4H_2_O and 18.25 g FeCl_3_·6H_2_O were mixed and dissolved in 150 mL distilled water. The solution was stirred while 50 mL of NaOH were slowly added. Solution was stirring until its color changed from light brown to black. Fe_3_O_4_ nanoparticles were washed by centrifugation and redispersed in distilled water three times. Then, 1.5 g sodium oleate was dissolved in 50 mL distilled water and added to the Fe_3_O_4_ nanoparticles under vigorous stirring for 30 min. The excess surfactant was removed by dialysis. The dialysis membranes with MWCO 3500 (Spectrum Laboratories, Inc., Rancho Dominguez, CA, USA) to dialyze the NPs were used, and, in principle, all unbound surfactant molecules would be removed.

### 4.2. Characterization of Nanoparticles

Fe_3_O_4_ NPs were analyzed for phase composition by the X-ray powder diffraction over the 2q range from 20 to 70° at a rate of 1.5°/min, using Cu-Ka radiation. The morphology and size distribution of the Fe_3_O_4_ NPs were observed by a transmission electron microscope (TEM, JEOL, Tokyo, Japan). A Fourier-Transform infrared spectrometer (FT-IR; Perkin Elmer, Waltham, MA, USA) was used to analyze the surface composition of the NPs. The zeta potential of Fe_3_O_4_ NPs was measured by light scattering using the Delsa™ Nano Zeta Potential and Submicron Particle Size Analyzer (Beckman Coulter, Brea, CA, USA). The weight loss of the dried sample was monitored under N_2_ from 100 to 800 °C at a heating rate of 10 °C/min by a thermogravimetric analyzer (Perkin Elmer).

For AFM measurements, 20 µL of Fe_3_O_4_ nanoparticle solution was put on freshly cleaved mica. After 30 s, drops were removed from the surface of mica by spinning the sample (spin drying). AFM measurements were performed using AFM Innova (Veeco Inc., Plainview, NY, USA) in tapping mode. RTESP7 probes (tip radius < 10 nm) were used. Hydrodynamic particle diameter and zeta potential measurements were performed using particle size and zeta potential analyser ZetaPALS (Brookhaven Instruments Inc., Holtsville, NY, USA).

### 4.3. Cell Culturing

Immortalized mouse embryonic fibroblast cell line NIH3T3 was purchased from American Type Culture Collection. Cells were cultured in cell growth medium (DMEM; Gibco, Waltham, MA, USA), supplemented with 10% (*v*/*v*) fetal bovine serum (FBS) (Gibco), 100 U/mL penicillin and 100 mg/mL streptomycin. Cells were maintained at 37 °C in a humidified atmosphere containing 5% of CO_2_. The cells were routinely subcultured 2–3 times a week in 25 cm^2^ culture dishes.

### 4.4. Treatment of Cells with Nanoparticles

For intracellular imaging studies, cells were seeded into an 8-chambered cover glass plate (Nalge Nunc International, Rochester, NY, USA) with a density of 3 × 10^4^ cells/chamber and subsequently incubated at 37 °C in a humidified atmosphere containing 5% of CO^2^ for 24 h. For the Fe_3_O_4_ uptake dynamics and intracellular localization evaluation, cells were treated with 65 ng/mL of Fe_3_O_4_ nanoparticles and incubated for the next 1, 6, 24, 48 or 72 h. Before imaging of nanoparticles, cells were fixed. After removing the growth media with nanoparticles, cells were washed 3 times with 7.4 pH phosphate buffered saline (PBS) (Gibco) adding 200 µL to each well. Cells were fixed by treating them for 15 minutes with sufficient amount of 4% paraformaldehyde (Sigma-Aldrich, St. Louis, MO, USA). Then, the cells were washed again with PBS three times.

### 4.5. Visualization and Imaging of Fe_3_O_4_ in Cells

For iron visualization, a Prussian Blue Cell Staining Reagent Pack (Sigma-Aldrich) was used. It consists of two reagents: hydrochloric acid and potassium ferrocyanide. Equal amounts of both reagents were mixed together and added to each well with cells. Fixed cells were treated with working solution for 15 min. During the reaction, any ferric ion present in the cells combines with the ferrocyanide and results in the formation of a bright blue pigment called Prussian blue, or ferric ferrocyanide. Then, the working solution was aspirated and the fixed cells were washed with PBS. The accumulation of nanoparticles in cells was observed using Nikon Eclipse TE2000 (Nikon, Tokyo, Japan) bright-field microscope equipped with a digital color camera Leica DFC290. To gain a better insight on cell structure, we also used differential interference contrast method (DIC).

### 4.6. Cytotoxicity Measurements

XTT cell viability assay was done to analyze toxicity of SPIONs. It is based on the measurement of mitochondrial enzymes activity in viable cells that reduce XTT, which is a tetrazolium derivative. XTT reduction is proportional to the number of viable cells in the sample and can be photometrically quantified at 490 nm [34]. The cells were seeded on a 96-wellplate (BD Falcon, San Jose, CA, USA) at a density of 1.5 × 10^4^ cells/well and incubated for 24 h before the nanoparticles were applied. The old medium was replaced with fresh medium containing nanoparticles, while media alone without nanoparticles was a control. After treatment, the old medium with nanoparticles was carefully aspirated and the cells were washed three times with DPBS (pH 7.0) (Sigma-Aldrich) before 100 μL of growth media were added to each well. To prepare a reaction solution sufficient for one plate (96 wells), 0.1 mL activation solution (*N*-methyl dibenzopyrazine methyl sulfate) (Biological Industries, Kibbutz Beit-Haemek, Israel) and 5 mL XTT reagent (Biological Industries) were mixed. Then, 50 μL of the reaction solution were added to each well and the plate was incubated in an incubator at 37 °C. After another 4 h of incubation, optical density values at 490 nm were measured using the microplate reader (BioTek, Winooski, VT, USA). After obtaining values of absorbance, they were recalculated as percentage values of viability. Absorbance value of control group was equated to 100% and the rest of the values were calculated proportionally to control.

### 4.7. MR Imaging

In vitro. All MR imaging examinations were performed by using a clinical 1.5 T MR scanner (Philips Achieva, Philips Medical Systems, Best, The Netherlands) with a Sense Flex-M coil (Philips Medical Systems). SPION aqueous solutions were placed in a series of 2 mL plastic Eppendorf test tubes (Sigma-Aldrich) with concentrations varying from 1300 to 0.13 mg/L for imaging. Tubes containing the SPION solutions were arrayed in order of concentration and tube containing water was placed as control. The MR images in vitro were acquired using T2-weighted turbo spin-echo (T2W-TSE) imaging sequence with the following parameters: scanning plane—coronal, repetition time (TR) 1800 ms, echo time (TE)—60 ms, matrix size—256 × 256; field of view (FOV)—150 × 150 mm, slice thickness—2.0 mm, number of acquisitions (NSA)—6. Acquired images were analyzed in order to examine the MR signal intensity (SI) versus the Fe_3_O_4_ concentration. Quantitative MR imaging analysis was performed and MR SI values were measured by drawing regions of interest (ROIs) of 57 mm^2^ over test tubes sliced images using Siemens Syngo workstation’s MI Apps software (Software version: 8.5.10.10 SP3; Siemens Healthcare GmbH, Erlangen, Germany) and calculated in relative units.

In vivo. MRI in vivo examinations were performed by using the same equipment as for in vitro studies. In imaging in vivo experiments, anesthesia is necessary in order to ensure the constant restraint of the animals. The animals were anesthetized with an intramuscular injection of 0.1 mL of ketamine hydrochloride per 100 g of body weight. During the MR imaging, it was additionally administrated up to 0.2 mL of ketamine hydrochloride per 100 g of body weight. One imaging session continues up to 3 h. Wistar rats (weighting 200–250 g) were anesthetized and imaged first (as control) and later at different time points (in a period of 2 months) after the intramuscular injection of Fe_3_O_4_ nanoparticles solution in the left hind paw. Two different concentrations of the Fe_3_O_4_ nanoparticles solution (in volume of 0.2 mL) were chosen for administration: 520 µg Fe_3_O_4_/kg (SPION-high dose) and 20.8 µg Fe_3_O_4_/kg (SPION-low dose). In addition, physiological saline (0.9% NaCl) was administrated in the right hind paw for the reference. In vivo T2*-weighted MR images were acquired using fast gradient-echo (T2W-FFE) MR imaging sequence with the following parameters: scanning plane—coronal, repetition time (TR) 2000 ms, echo time (TE)—16.12 ms, matrix size—256 × 256; field of view (FOV)—127 × 35 mm, slice thickness—2.0 mm, number of acquisitions (NSA)—4. The acquired images were analyzed in order to evaluate migration of Fe_3_O_4_ nanoparticles from the injection site. The MR SI values were obtained by drawing ROIs over the injection site and the background tissue in coronal images using Philips Dicom Viewer software (R3.0-SP3, Philips Medical Systems) and calculating the MR signal relative intensity.

### 4.8. Animal Models

The healthy adult Wistar rats, 6–8 weeks old and 200–250 g weight, were obtained from State Research Institute Centre for Innovative Medicine, Vilnius, Lithuania.

All animals were maintained at a constant temperature (22 ± 1 °C), relative humidity (55% ± 10%) and photoperiod (12 h light/dark cycle) in the Open Access Centre at National Cancer Institute, Vilnius, Lithuania. The animals were fed standard rodent chow and purified water ad libitum. All animals’ procedures were performed in accordance with the guidelines established by the Lithuanian Care Committee which approved the study (No.Gd-29).

## 5. Conclusions

This study provides some initial evidence that SPIONs accumulate in NIH3T3 cells and they are nontoxic for cells, according to a standard XTT test. Our findings demonstrate significant signal reduction with increasing of Fe_3_O_4_ concentration in the T2-weighted MRI. Moreover, low concentration of SPIONs was completely eliminated from the injection site after three weeks. Our methods utilized for visualization of SPIONs showed that these nanoparticles could be used as dual cancer imaging: for MRI as contrast agents and for traditional optical biopsy in morphology using Prussian Blue staining.

## Abbreviations

SPIONs	Superparamagnetic iron oxide nanoparticles
MNPs	Magnetic nanoparticles
MRI	Magnetic resonance imaging
